# Scattering symmetry-breaking induced spin photocurrent from out-of-plane spin texture in a 3D topological insulator

**DOI:** 10.1038/s41598-020-67612-3

**Published:** 2020-06-30

**Authors:** Y. Q. Huang, I. A. Buyanova, W. M. Chen

**Affiliations:** Department of Physics, Chemistry and Biology, Linköping University, 581 83 Linköping, Sweden

**Keywords:** Topological insulators, Spintronics

## Abstract

We theoretically study helicity-dependent photocurrent in a three-dimensional topological insulator Bi_2_Te_3_ under elastic scattering of different symmetries. By exploring spin-selective optical transitions and symmetry-breaking scattering, we are able to address the out-of-plane spin texture of the topological helical surface states and to generate directional, spin-polarization tunable photocurrent that is otherwise forbidden for the original C_3v_ symmetry of the surface. This can be achieved regardless of the Fermi level, even under the condition when the topological states are inaccessible in dark. This work paves the way to robustly explore the out-of-plane spin texture for harvesting opto-spintronic functionalities of topological insulators.

A three-dimensional (3D) topological insulator (TI), for instance Bi_2_Te_3_, has an insulating bulk and a metallic surface that is characterized by the topological surface states (TSSs)^1–9^. Owing to the strong spin–orbit interaction, the spin of the TSS is locked to its momentum such that efficient electron spin polarization as well as non-equilibrium spin current can be generated electrically, making it attractive for dissipationless spintronic and quantum information applications^10–18^. Recently, an alternative method is discovered through which spin-polarized current can be efficiently created in 3D TIs by circularly polarized light excitation^19–25^. This so-called circular photogalvanic effect allows for a non-equilibrium flow of spin current and exhibits large tolerance against doping conditions of the materials^19,26–28^. The high efficiency and flexibility of the method not only suggest great advantage for studying spin generation, manipulation and transport in TIs, but also open a new gateway for future opto-spintronics.

The early studies on spin and non-equilibrium spin current generation in 3D TI have focused on the spin component that is parallel to the surface of the 3D TI. In materials like Bi_2_Te_3_, however, a strong hexagonal warping effect also leads to a pronounced out-of-plane spin texture of the TSS that rises up to the strength of its in-plane counterpart and progressively increases with the energy of the TSS moving away from the Dirac point. In this work, we theoretically investigate the generation of spin polarization and non-equilibrium spin current through such out-of-plane spin texture by exploring the symmetry-breaking effect induced by scattering. We show that elastic scattering of the TSS via line defects breaks the C_3v_ symmetry of the Bi_2_Te_3_ surface and consequently gives rise to a new photocurrent component, of which the magnitude and spin polarization can be tuned by changing the Fermi energy.

The device structure and measurement geometry used in this work is defined in Fig. 1(a). We consider a (111)-grown Bi_2_Te_3_ thin film made of 30 quintuple layers (QLs). The x- and z-axis are chosen to lie along the $[\stackrel{-}{1}10]$ and $[111]$ axes of the film, respectively. The device is illuminated with single-color circularly polarized light propagating in the x–z plane with an incidence angle $\theta$. The resulting photocurrent density under the ${\sigma }^{+}$ or ${\sigma }^{-}$ polarized excitation, ${{\varvec{j}}}^{\sigma \pm }$, is decomposed into components along the x and y directions, marked by the yellow arrows in Fig. 1(a). The helicity-dependent photocurrent (HPC) density ${{\varvec{j}}}^{pol}$ is the photocurrent density difference taken between the two opposite excitation helicities. The tight-binding description of the electronic structure of the Bi_2_Te_3_ thin film is adopted from Ref.^29^ by considering the Bi_2_Te_3_ thin film as stacking of N number of layers of Bi and Te atoms which are arranged in a 2D hexagonal lattice in the x–y plane. The preserved translation symmetry in the x–y plane gives rise to the projected 2D Brillouin zone as shown in Fig. 1(a). Only one equivalent atom needs to be considered for each layer. Along the z direction, the equivalent atoms are arranged with the repeating unit of Te2–Bi–Te1–Bi–Te2, known as the quintuple layer (QL) shown in Supplementary Fig. S1(a). By considering this “chain” of atoms, one can write down the Hamiltonian for the (111)-grown Bi_2_Te_3_ thin film with an arbitrary number of atomic layers and different termination atoms,

**Figure 1:**
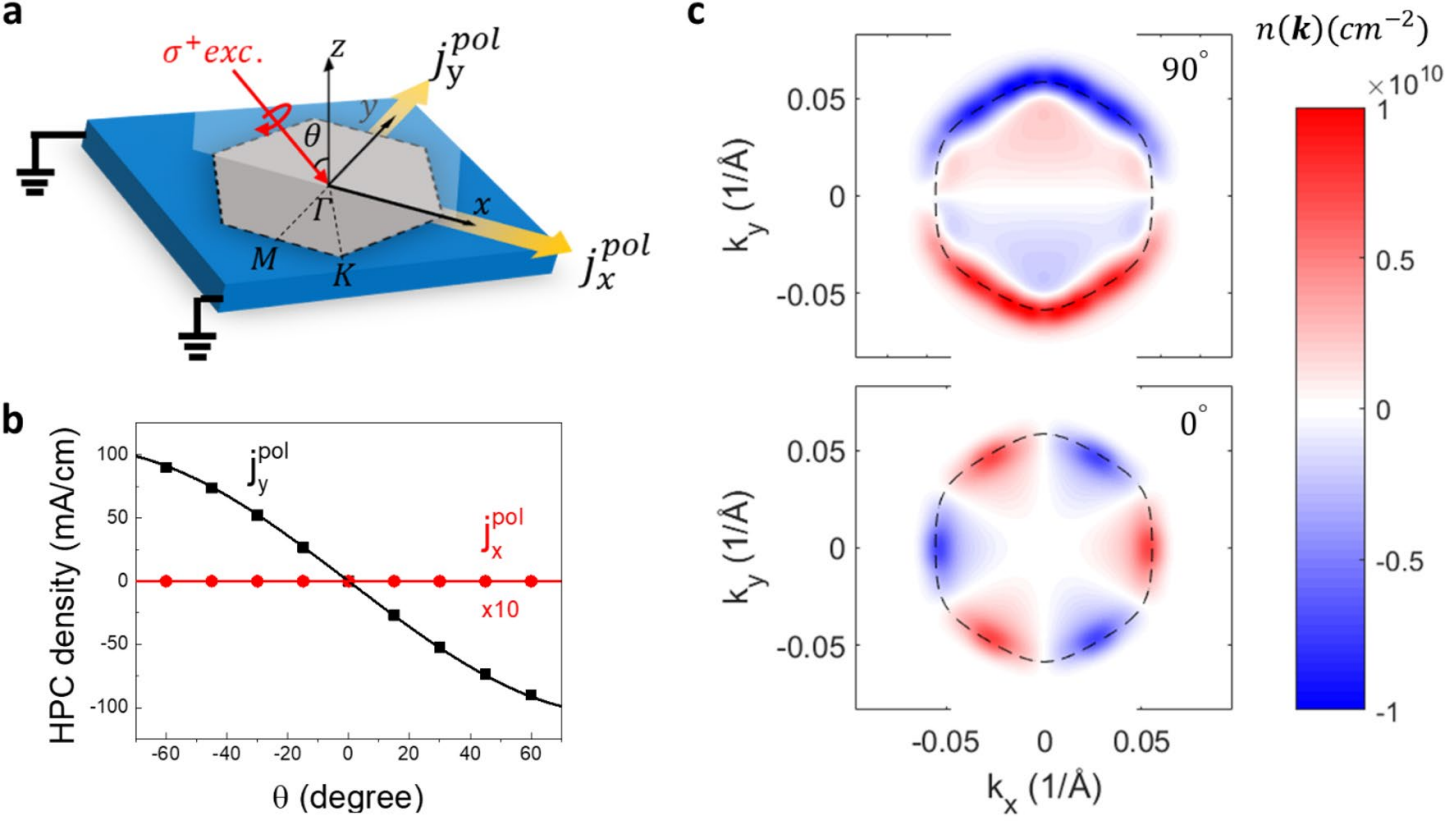
HPC component and spin-selective optical transitions. (**a**) The device structure used in the calculations. The projected 2D Brillouin zone is also shown with respect to the measurement frame. (**b**) The calculated ${j}_{x}^{pol}$ and ${j}_{y}^{pol}$ as a function of the incidence angle $\uptheta$ of the excitation light as defined in (**a**). The ${E}_{F}$ is set to be at 192 meV above the Dirac point and the excitation photon energy is at 1.25 eV. (**c**) The helicity-dependent steady-state photocarrier distribution $n\left({\varvec{k}}\right)$ for the upper brunch of the Dirac cone, calculated for the grazing ($\uptheta ={90}^{^\circ }$, upper panel) and normal ($\uptheta ={0}^{^\circ }$, lower panel) incidence. The Fermi circle is marked by the dashed line in each case.

1 $${\mathcal{H}}_{0} = \mathop \sum \limits_{{ii^{\prime},\alpha \alpha ^{\prime},\sigma }} t_{{ii^{\prime}}}^{{\alpha \alpha ^{\prime}}} e^{{i\user2{k} \cdot \left( {\user2{r}_{i} - \user2{r}_{{i^{\prime}}} } \right)}} c_{{i\alpha \sigma }}^{\dag } c_{{i^{\prime}\alpha ^{\prime}\sigma }} + \mathop \sum \limits_{{i,\alpha \alpha ^{\prime},\sigma \sigma ^{\prime}}} i\alpha \sigma {\text{|}}\lambda _{i} \user2{L} \cdot \user2{S}{\text{|}}i\alpha ^{\prime}\sigma ^{\prime}c_{{i\alpha \sigma }}^{\dag } c_{{i\alpha ^{\prime}\sigma ^{\prime}}}$$

Here, $i\left({i}^{^{\prime}}\right)$ labels the atom sites. $\alpha \left({\alpha }^{^{\prime}}\right)$ and $\sigma \left({\sigma }^{^{\prime}}\right)$ represent the atomic orbitals $\left(s,{p}_{x},{p}_{y},{p}_{z}\right)$ and spin quantum number, respectively. ${c}_{i\alpha \sigma }^{\dag}\left({c}_{i\alpha \sigma }\right)$ is the electron creation (annihilation) operator, which creates (destroys) the electron with spin of $\sigma$ and atomic orbital of $\alpha$ at site $i$. ${\lambda }_{i}$ is the spin–orbit strength at site $i$. The first term in Eq. (1) includes the on-site self-energy ($i={i}^{^{\prime}}$) and the hopping energy between the nearest/second/third neighbor site ($i\ne {i}^{^{\prime}}$), which is parameterized by the overlap integral ${t}_{i{i}^{^{\prime}}}^{\alpha {\alpha }^{^{\prime}}}$ between the $\alpha$ orbital at site $i$ and ${\alpha }^{^{\prime}}$ orbital at site ${i}^{^{\prime}}$. The second term is the on-site spin–orbit interaction, which is the key ingredient for TSS formation and the driven force of CPGE in TI.

The electronic band structure of the Bi_2_Te_3_ thin film can be obtained after diagonalizing the above Hamiltonian. The results are shown in Supplementary Fig.S1(b) with the tight-binding parameter from Ref.^29^. The dispersion relation quantitatively reproduces the previous results from the angular resolved photoemission spectroscopy (ARPES) and various theoretical reports^30–33^. The TSS is automatically obtained, evident from the appearing of the Dirac cone in the bulk bandgap.

The photocurrent ${{\varvec{j}}}^{\sigma \pm }$ is determined by the steady-state density matrix ${\rho }^{\sigma \pm }$ of TSS as ${{\varvec{j}}}^{\sigma \pm }=-e\mathrm{T}\mathrm{r}\left[{\rho }^{\sigma \pm }{\varvec{v}}\right]$, where ${\varvec{v}}=\frac{1}{\hslash }{\nabla }_{{\varvec{k}}}{\mathcal{H}}_{0}$ is the velocity operator with ${\mathcal{H}}_{0}$ being the tight-binding Hamiltonian. ${\rho }^{\sigma \pm }$ is obtained from the steady-state solution of the dynamic equation of the form:

2 $$\frac{d{\rho }^{\sigma \pm }}{dt}=\frac{1}{i\hslash }\left[{\mathcal{H}}_{0},{\rho }^{\sigma \pm }\right]+{G}^{\sigma \pm }-{\gamma }^{rlx}\left({\rho }^{\sigma \pm }-{\rho }^{(0)}\right)$$

With the basis that diagonalizes ${\mathcal{H}}_{0}$, we constrain the calculations only to the diagonal term of ${\rho }^{\sigma \pm }$. By doing so, we ignore excitation of superposition states of TSS, which is reasonable for the TSS states with a fast decoherent process. The second term generates carriers in the Dirac cone following the spin selective optical transitions between TSS $\left|{\varvec{k}}\rangle \right.$ and bulk state $\left|n,{\varvec{k}}\rangle \right.$:

3 $${G}_{{\varvec{k}}}^{\sigma \pm }=\frac{1}{{\mathrm{\hslash }}^{2}}{\sum }_{n}{\left|\langle n,{\varvec{k}}|-e{{\varvec{A}}}^{\pm }\cdot {\varvec{v}}|{\varvec{k}}\rangle \right|}^{2}\left[\delta \left({E}_{\varvec{k}}-{E}_{n,\varvec{k}}-\mathrm{\hslash }\omega \right)\left(1-{\rho }^{(0)}\right)-\delta \left({{E}}_{\mathrm{n},\varvec{k}}-{{E}}_{\varvec{k}}-\mathrm{\hslash }\upomega \right){\rho }^{(0)}\right]$$

The summation runs through all the bulk states with the same ${\varvec{k}}$. ${{\varvec{A}}}^{\pm }$ is the Fourier transformed vector potential. ${\rho }^{(0)}\equiv\Theta \left({E}_{F}-{E}_{{\varvec{k}}}\right){\delta }_{{\varvec{k}},{{\varvec{k}}}^{\boldsymbol{^{\prime}}}}$ describes the equilibrium distribution at 0 K, in which $\Theta \left(x\right)$ is the step function, ${E}_{F}$ and ${E}_{{\varvec{k}}}$ are the Fermi energy and eigen energy of the TSS, respectively. The last term in Eq. (2), ${\gamma }^{rlx}\equiv \frac{{\delta }_{{\varvec{k}}{{\varvec{k}}}^{^{\prime}}}}{{T}_{1}}$, describes the relaxation of the photogenerated carriers within the TSS. The carrier decay rate, ${T}_{1}^{-1}={\tau }_{1}^{-1}+{\tau }_{2}^{-1}\cdot \frac{{E}_{{\varvec{k}}}-{E}_{F}}{{k}_{B}{T}_{eff}\left({e}^{-\frac{{E}_{{\varvec{k}}}-{E}_{F}}{{k}_{B}{T}_{eff}}}+1\right)}$, includes energy independent and dependent contributions that describe the recombination and thermalization process^34,35^. Here, ${\tau }_{2}\equiv \frac{1}{{T}_{eff}}\frac{d{T}_{eff}}{dt}$ defines the rate of thermalization process. We noted that ${G}_{{\varvec{k}}}^{\sigma \pm }$ is essential the driving term of the kinetic equation, which is balanced by the relaxation terms and leads to the steady-state density matrix. In the presentation of TSS $\left|{\varvec{k}}\rangle \right.$, ${G}_{{\varvec{k}}}^{\sigma \pm }$ only contains diagonal contribution. This is the result of first-order perturbation theory by considering weak optical excitation^36^. Immediately following this simplification, the relaxation terms only need to include diagonal contribution which is represented by ${\gamma }^{rlx}$, whereas the off-diagonal inter-band transition contribution is out of scope of current work^37^. However, we do noted that in the presence of strong excitation where higher order perturbation is need, such effect will need to be in place.

In Fig. 1(b), we calculate ${j}_{x }^{pol}$ and ${j}_{y }^{pol}$ as a function of $\theta$. The excitation photon energy is fixed at 1.25 eV with 60-meV broadening and the relaxation parameter ${\tau }_{1}=25 ps$. ${k}_{B}{T}_{eff}=4 meV$ is chosen to represent the thermalization process observed in the angular resolved photoemission spectroscopy (ARPES) experiments^35^. The ${E}_{F}$ is fixed at 192 meV above the Dirac point. We note that the TSSs involved in ${\rho }^{\sigma \pm }$ are selected from the top surface of the film with the sampled k-point mesh as described in Supplementary Note 1. The exclusion of the contribution from the bottom TSSs breaks the inversion symmetry, which is a necessary condition to observe any HPC. This also qualitatively simulates the experimental condition: the incidence light is predominantly absorbed or reflected at the top surface of the films. The calculated results represent the typical experimental observation that a tilted excitation ($\uptheta \ne {0}^{^\circ }$) creates an HPC propagating perpendicular to the incidence plane (${j}_{y }^{pol}$ in our configuration). For $\uptheta ={0}^{^\circ }$, no HPC is detected along either x or y directions. In the symmetry argument put forward by McIver et.al., the ${C}_{3v}$ symmetry of the Bi_2_Te_3_ surface forbids HPC under the normal incidence condition^19,38^. Microscopically, this is explained in the calculated helicity-dependent steady-state photocarrier distribution $n\left({\varvec{k}}\right)=\langle {\varvec{k}}|{\rho }^{\sigma +}|{\varvec{k}}\rangle -\langle {\varvec{k}}|{\rho }^{\sigma -}|{\varvec{k}}\rangle$ for the TSSs shown in Fig. 1(c). The upper and lower panel of Fig. 1(c) correspond to the grazing ($\uptheta ={90}^{^\circ }$) and normal ($\uptheta ={0}^{^\circ }$) incidence condition. The Fermi surface at equilibrium intersects the TSS at ${E}_{{\varvec{k}}}={E}_{F}$, which is marked by the dashed curve in each case. The Fermi circle develops a hexagonal shape, suggesting a strong modification of the k-linear dispersion relation of a massless Dirac fermion due to the hexagonal wrapping effect. Under the grazing incidence, $n({\varvec{k}})$ develops anti-symmetric patterns along the k_y_ axis. The imbalanced distribution of the photocarriers contributes to a net current flow along the -y direction, which explains the origin of ${j}_{y }^{pol}$ shown in Fig. 1(b). Despite the complex pattern of $n({\varvec{k}})$, which is due to optical transitions involving different bulk bands, $n({\varvec{k}})$ shows a correlation with the in-plane spin texture component $\langle {S}_{x}\rangle \equiv \langle {\varvec{k}}|{S}_{x}|{\varvec{k}}\rangle$ of the TSSs. $\left|n({\varvec{k}})\right|$ is found to be at the maximum (or zero) along the k_y_ (or k_x_) axis where $\left|\langle {S}_{x}\rangle \right|$ peaks (or vanishes). This is also seen in Fig. 1(b) that ${j}_{y }^{pol}$ can be nicely fitted with ${j}_{y }^{pol}\propto sin\theta$, suggesting that the photocurrent scales with the projection of the photon angular momentum along the in-plane spin texture. Under the normal incidence condition, in contrast, $n({\varvec{k}})$ follows the out-of-plane spin texture component $\langle {S}_{z}\rangle$, showing a three-fold alternating behavior around the Dirac point. Along the $\mathrm{K}-\Gamma -\mathrm{K}$ (or $\mathrm{M}-\Gamma -\mathrm{M}$) direction, both $\left|n({\varvec{k}})\right|$ and $\left|\langle {S}_{z}\rangle \right|$ reach their maxima (or zeros). Although under both incidence conditions the photocurrent density generated by the spin-dependent optical transitions is comparable thanks to a pronounced $\left|\langle {S}_{z}\rangle /\langle {S}_{x}\rangle \right|$ ratio within the given k-space, the C_3v_ symmetry restricts the $\langle {S}_{z}\rangle$, hence the $n({\varvec{k}})$ under the normal incidence condition, to be canceled out over the k-space integration. Therefore, no HPC is expected from the contribution of $\langle {S}_{z}\rangle$.

The key to harvesting the contribution of $\langle {S}_{z}\rangle$ and creating non-equilibrium spin current with finite out-of-plane spin polarization is to break the surface symmetry. Here, we consider the effect of elastic scattering by a scattering potential. The elastic scattering introduces an additional contribution to the relaxation term in ${\gamma }^{rlx}$,

4 $${\gamma }_{{\varvec{k}},{{\varvec{k}}}^{\boldsymbol{^{\prime}}}}^{elastic}={W}_{{\varvec{k}},{{\varvec{k}}}^{\boldsymbol{^{\prime}}}}-{\delta }_{{\varvec{k}},{{\varvec{k}}}^{\boldsymbol{^{\prime}}}}\sum_{{{\varvec{k}}}^{\boldsymbol{^{\prime}}\boldsymbol{^{\prime}}}}{W}_{{\varvec{k}},{{\varvec{k}}}^{\boldsymbol{^{\prime}}\boldsymbol{^{\prime}}}}$$

Here, ${W}_{{\varvec{k}},{{\varvec{k}}}^{\boldsymbol{^{\prime}}}}=\frac{2\pi }{\hslash }{{n}_{i}\left|\langle {\varvec{k}}|{V}_{s}\left({\varvec{r}}\right)|{{\varvec{k}}}^{\boldsymbol{^{\prime}}}\rangle \right|}^{2}\delta ({E}_{n{\varvec{k}}}-{E}_{m{{\varvec{k}}}^{\boldsymbol{^{\prime}}}})$ is the elastic scattering rate between the given TSSs. ${V}_{s}\left({\varvec{r}}\right)$ is the scattering potential. ${n}_{i}$ is the concentration of the defects. To explore a symmetry-breaking effect, we consider two representative scattering potentials, namely point scatters (${V}_{s}\left({\varvec{r}}\right)={V}_{0}\delta ({\varvec{r}})$) and line defects along the y axis (${V}_{y}\left({\varvec{r}}\right)\equiv {V}_{0}\delta \left(x\right)$). While the point scatters maintain the original surface symmetry, the line scatters lower the symmetry to ${C}_{s}$.

The effect of different scattering centers is presented by the scattering matrix element, which in general can be expanded with the tight-binding basis as:

5 $$\langle {{\varvec{k}}}^{\boldsymbol{^{\prime}}\boldsymbol{^{\prime}}}|{V}_{s}({\varvec{r}})|{{\varvec{k}}}^{\boldsymbol{^{\prime}}}\rangle ={\sum }_{i\alpha \sigma }{c}_{i\alpha \sigma }^{{{\varvec{k}}}^{\boldsymbol{^{\prime}}\boldsymbol{^{\prime}}}*}{c}_{i\alpha \sigma }^{{{\varvec{k}}}^{\boldsymbol{^{\prime}}}}\frac{1}{{(2\pi )}^{2}}\int {\stackrel{\sim }{V}}_{s}\left({\varvec{k}}\right)\delta [\varvec{a}_{1}\cdot \left({\varvec{k}}-\Delta {\varvec{K}}\right)]\delta [\varvec{a}_{2}\cdot \left({\varvec{k}}-\Delta {\varvec{K}}\right)]{d}^{2}{\varvec{k}}$$

Here ${c}_{i\alpha \sigma }^{{\varvec{k}}}$ is the expanding coefficient for the TSS $\left|{\varvec{k}}\rangle \right.={\sum }_{i\alpha \sigma }{c}_{i\alpha \sigma }^{{\varvec{k}}}{e}^{i{\varvec{k}}\cdot {{\varvec{r}}}_{i}}\left|i\alpha \sigma \rangle \right.$. $i$, $\alpha$ and $\sigma$ label the atomic site, orbit and spin of the localized states, respectively. ${\stackrel{\sim }{V}}_{s}\left({\varvec{k}}\right)$ is the Fourier transformation of ${V}_{s}({\varvec{r}})$. $\Delta {\varvec{K}}={{\varvec{k}}}^{\boldsymbol{^{\prime}}\boldsymbol{^{\prime}}}-{{\varvec{k}}}^{\boldsymbol{^{\prime}}}$ represents the momentum mismatch between the incoming and scattered TSSs. Equation (5) suggests that the scattering amplitude is finite when $\Delta {\varvec{K}}$ can be compensated by the scattering potential. It also requires finite overlap between the incoming and scattered TSS states, namely $\langle {{\varvec{k}}}^{\boldsymbol{^{\prime}}\boldsymbol{^{\prime}}}|{{\varvec{k}}}^{\boldsymbol{^{\prime}}}\rangle \ne 0$. In Fig. 2(a) and (b), we plot the steady-state $n({\varvec{k}})$ for the two types of scatters at $\uptheta ={0}^{^\circ }$. The point scatter preserves the rotational symmetry and the scattering is therefore expected to scale with ${\left|\langle {{\varvec{k}}}^{\boldsymbol{^{\prime}}\boldsymbol{^{\prime}}}|{{\varvec{k}}}^{\boldsymbol{^{\prime}}}\rangle \right|}^{2}$. For strict backscattering with ${{\varvec{k}}}^{{^{\prime}}{^{\prime}}}=-{{\varvec{k}}}^{^{\prime}}$, the initial and final state of the elastic scattering correspond to a Kramer pair linked by the time-reversal symmetry. ${W}_{{{\varvec{k}}}^{{^{\prime}}{^{\prime}}},{{\varvec{k}}}^{^{\prime}}}$ vanishes due to the orthogonality of the wavefunctions. Although strict backscattering is forbidden, elastic scattering is allowed for ${{\varvec{k}}}^{{^{\prime}}{^{\prime}}}\ne -{{\varvec{k}}}^{\boldsymbol{^{\prime}}}$^6,7,39^. Particularly in the current case of Bi_2_Te_3_, the strong hexagonal warping effect leads to the alternating out-of-plane spin texture, promoting the scattering across the hexagonal edge as shown by the arrows in Fig. 2(a). Since the scattering vectors join the TSSs with similar photocarrier populations, $n({\varvec{k}})$ is largely unaffected as compared with that in Fig. 1(c), which explains the similar behavior of the HPC. In contrast, the line defects scatter carriers anisotropically. While $\Delta {\varvec{K}}$ along the x direction is accommodated by the scattering potential, $\Delta {\varvec{K}}$ along the orthogonal direction needs to be conserved after the scattering. As a result, the scattering events are mainly carried out following the solid arrows in Fig. 2(b). The dashed arrow marks the scattering vector that is allowed by the potential but forbidden by ${\left|\langle {{\varvec{k}}}^{\boldsymbol{^{\prime}}\boldsymbol{^{\prime}}}|{{\varvec{k}}}^{\boldsymbol{^{\prime}}}\rangle \right|}^{2}=0$. The scattering vectors in Fig. 2(b) (the solid arrows) connect the TSSs with different populations and thus lead to a redistribution of the photocarriers. This breaks the three-fold rotation symmetry and generates a primary carrier imbalance along the k_x_ axis and consequently a new HPC component along the x axis. In Fig. 2(c) and (d) we plot the angular distribution of the photocarrier in the k-space for point and line scatters, respectively. The angle ${\theta }_{k}$ is the angle in the k-space and is defined counter-clockwise with respect to the k_x_ axis, as shown in Fig. 2(a) and (b). In both cases, $n({\theta }_{k})$ reach local extrema (or zeros) at the angles corresponding to the $\Gamma -\mathrm{K}$ (or $\Gamma -\mathrm{M}$) directions. For line scattering, an excess population of electrons and holes are found at ${\theta }_{k}={0}^{^\circ }$ and ${\theta }_{k}={180}^{^\circ }$. We note that, in another aspect, the resulting $n({\theta }_{k})$ is also a direct outcome of the topological protection of the TSSs that the scattering between the TSSs located at ${\theta }_{k}={0}^{^\circ }$ and ${\theta }_{k}={180}^{^\circ }$ are forbidden.

**Figure 2:**
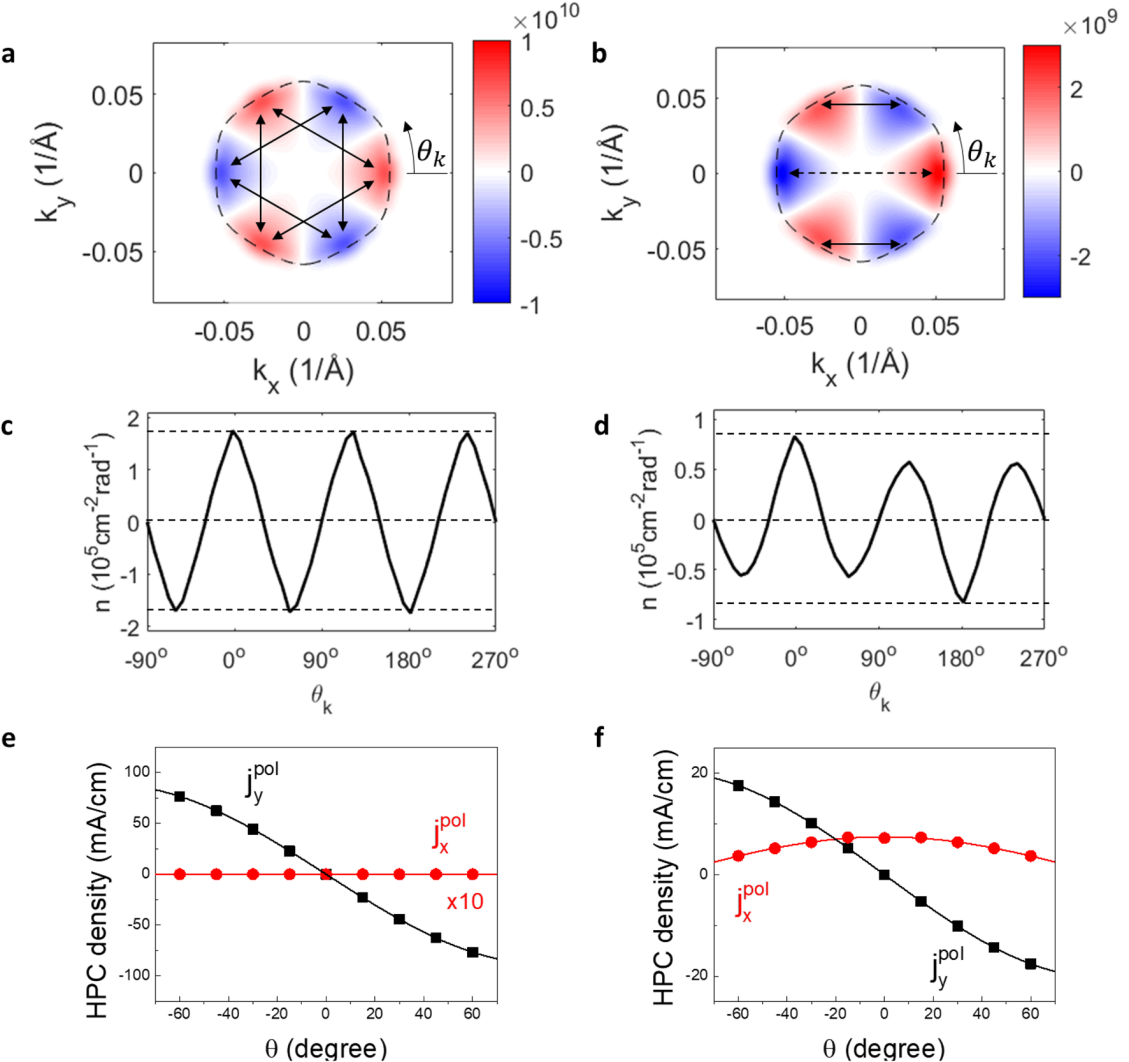
Scattering symmetry-breaking induced HPC. (**a**) and (**b**) are the $n\left({\varvec{k}}\right)$ calculated involving the elastic scattering from the point and line scatters, respectively. The featured scattering vectors are marked by the arrows and discussed in the main text. (**c**) and (**d**) are the angular distribution of the photocarriers in the k-space for the point and line potential scattering, respectively. ${\theta }_{{{k}}}$ is the orientation angle in the k-space as defined in (**a**, **b**). (**e**) and (**f**) are real-space angular dependence of the HPC components for the point and line scattering, respectively.

In Fig. 2(e) and (f), we compute the HPC density as a function of $\uptheta$ for the two types of scattering centers. The scattering potential and defect concentration are set at 1.6 $eV\cdot {\AA }^{2}$ and ${10}^{12}{ \mathrm{c}\mathrm{m}}^{-2}$^40^, respectively. The angular dependence of the HPC in the presence of point scatters is similar to that in Fig. 1(b) where the elastic scattering is absent. ${j}_{x}^{pol}$ remains vanished at all incidence angles, which is consistent with the symmetry constraint. In sharp contrast, for line scatters, ${j}_{x}^{pol}$ develops a finite value, which reaches the maximum at $\uptheta ={0}^{^\circ }$. The angular dependence of the new HPC component can be nicely fitted by the $\mathrm{c}\mathrm{o}\mathrm{s}\uptheta$ function as shown in Fig. 2(f), which represents the angular dependence of the projection of the photon angular momentum to the orientation of the out-of-plane spin texture. Although the computed results are for short-range potentials represented by the delta function, the symmetry consideration should also apply to other types of defects. As a simple test, in Supplementary Note 2, we have considered another two types of scattering potentials, namely the screened Coulomb potential and spin–orbit coupled scattering potential. We have computed and shown the results for $n({\varvec{k}})$ and angular dependence of HPC in Supplementary Fig. S3, which yields a consistent result showing that the new HPC vanishes due to the preserved surface symmetry in other types of scattering centers.

The new HPC component induced by the symmetry-breaking scattering offer new opportunities and a convenient and sensitive means not only to optically generate but also to detect the out-of-plane spin component under the normal incidence condition in a 3D TI. Here, opposite orientations of the out-of-plane spin component directly lead to opposite directions of the surface photocurrent. This extends the application of the extradentary spin-momentum locking of a 3D TI from only the commonly known in-plane spin component^19–23^ to the out-of-plane spin component that is more relevant to the majority of potential spintronic devices that employ interlayer spin transport. To evaluate the robustness of the approach, we calculate the symmetry-breaking scattering induced HPC as a function of the Fermi level. In Fig. 3(a), we show the results of the computed HPC by varying *E*_*F*_ from − 0.15 eV, when the Fermi level intersects with the bulk valence band at the vicinity of $\Gamma$ point (8 meV beneath the Dirac point), to 0.15 eV, when the *E*_*F*_ reaches the conduction band minimum. The Dirac point (DP) is located at − 0.142 eV. The tuning range of *E*_*F*_ covers both cases when the equilibrium Fermi level is inside and outside of the bulk bandgap. The calculation is done by considering ${\sigma }^{+}$ polarized excitation under the normal incidence condition. For the selected excitation photon energy of 1.25 eV, ${j}_{x}^{pol}$ maintains the positive sign and would generally be finite due to the aforementioned symmetry consideration regardless of the choice of ${E}_{F}$, which suggests the robustness of the proposed scheme to detect spin generation. As a test, we have calculated HPC at *E*_*F*_ = 0 eV and 0.15 eV where the Fermi level intersects with the bulk valence and conduction band respectively. The results are shown in Supplementary Fig. S2 for a line defect concentration of $1.0\times {10}^{11} {\mathrm{c}\mathrm{m}}^{-2}$. The angular dependence of the resulting HPC is similar to that in Fig. 2, which proves persistence of the new HPC through a large tuning range of ${E}_{F}$. Note that ${j}_{x}^{pol}$ can also be chosen to carry a different sign if the character of the bulk states involved in the optical transition alters by carefully selecting another excitation photon energy. The amplitude change of the ${j}_{x}^{pol}$ with changing ${E}_{F}$ represents the transitions involving different bands. It is also clearly seen in Fig. 3(a) that the magnitude of ${j}_{x }^{pol}$, proportional to the detection sensitivity, follows the change of the line-defect density ${n}_{i}$. The ${j}_{x }^{pol}$ increases by about two orders of magnitude when ${n}_{i}$ changes by the same orders of magnitude, i.e. from $1.0\times {10}^{9}$ to $1.0\times {10}^{11} {\mathrm{c}\mathrm{m}}^{-2}$. This close correlation confirms the important role of the symmetry-breaking scattering in HPC. Next, in Fig. 3(b), we show how the new HPC component reacts to the change of optical spin generation that follows the light helicities changes under the normal incidence condition. Here, we consider ${n}_{i}=1.0\times {10}^{11} {\mathrm{c}\mathrm{m}}^{-2}$ and ${E}_{F}=0.05 eV$. $\gamma$ is the phase different between the $x$- and $y$-component of the excitation polarization and the corresponding polarization states are also illustrated in Fig. 3(b). It is evident that ${j}_{x }^{pol}$ reverses the sign at $\gamma ={90}^{^\circ }$ and $\gamma ={270}^{^\circ }$, which corresponds to the optical spin generation with positive and negative helicities. At $\gamma ={180}^{^\circ }$, the excitation light becomes linearly polarized and no spin generation is detected (${j}_{x }^{pol}=0$) as the consequence of the vanishing excitation light helicity, again demonstrating that HPC is spin dependent.

**Figure 3:**
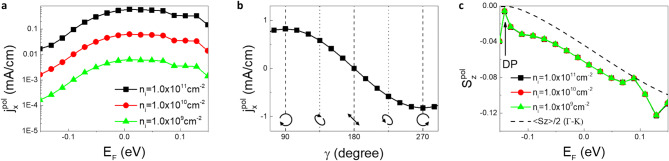
Detection of the out-of-plane spin component. (**a**) ${E}_{F}$ dependence of ${j}_{x}^{pol}$ for different densities of the line scatters. The calculation is performed for excitation photon energy of 1.25 eV under the normal incidence condition. The excitation light polarization is fixed at ${\sigma }^{+}$. (**b**) ${j}_{x}^{pol}$ as a function of different polarization states of the excitation light. $\gamma$ is the phase difference between the $x$- and $y$-components of the excitation polarization. The polarization states for different $\gamma$ are schematically illustrated. (**c**) Out-of-plane spin polarization ${S}_{z}^{pol}$ for the carriers contributing to HPC as a function of the Fermi energy, with different densities of the line scatter. The dashed line shows the energy dependence of the out-of-plane spin texture along the $\Gamma -\mathrm{K}$ axis in the k-space.

The spin polarization of the new HPC component is found to depend on the Fermi level, as shown in Fig. 3(c). By computing ${S}_{z}^{pol}=\mathrm{T}\mathrm{r}\left[\left({\rho }^{\sigma +}-{\rho }^{\sigma -}\right){S}_{z}\right]/\mathrm{T}\mathrm{r}\left[{\rho }^{\sigma +}-{\rho }^{\sigma -}\right]$, we demonstrate that the out-of-plane spin polarization of the photocarriers in the TSSs that contribute to the photocurrent can be adjusted by the Fermi level, which can for example be conveniently accomplished by applying gate voltage. The as-computed ${S}_{z}^{pol}$ can be tuned with ${E}_{F}$ following the energy dependence of the out-of-plane spin texture, which is shown by the dashed line in Fig. 3(c) regardless of the scattering centers density is altered by two order of magnitude. Such “universal” behavior of ${S}_{z}^{pol}$ is due to the fast thermalization process, namely the ${T}_{1}$ for TSSs in the vicinity of ${E}_{F}$ is substantially longer than that at the energies far away from the ${E}_{F}$. Since the linearized version of Eq. (2) gives $n\left({\varvec{k}}\right)\propto \left({G}^{\sigma +}-{G}^{\sigma -}\right){\left({\gamma }^{elastic}+{T}_{1}^{-1}\right)}^{-1}$, it follows that both the overall magnitude of $n\left({\varvec{k}}\right)$ and the relative contribution of ${\gamma }^{elastic}$ are maximized at the ${E}_{F}$. As long as this holds, the dominant contribution of the scattering-driven HPC component (${j}_{x}^{pol}$) is from the TSSs close to the ${E}_{F}$. When the ${E}_{F}$ is tuned through the bulk bandgap of the Bi_2_Te_3_, it intersects with TSSs and converts the out-of-plane spin texture of the states into spin-polarized photocurrent. It is important to note that symmetry-allowed HPC (${j}_{y}^{pol}$ at a tilted incident angle) does not “benefit” from such thermalization process. This is because the imbalanced distribution of photocarriers are associated with optical generation. Longer ${T}_{1}$ at the ${E}_{F}$ would make room for momentum relaxation that decreases the HPC.

These results suggest new ways to generate and control the spin photocurrent and its polarization in 3D TIs. By constructing specific scattering centers that break the C_3v_ symmetry of the Bi_2_Te_3_ surface, one can change both the magnitude and the spin polarization of the HPC. We note that, in the calculations, a simplified scattering potential is assumed, but the qualitative results should also work for more realistic defects: point defects like charged impurities and vacancies, or line defects such as atomic steps at edges of terraces, twin boundaries, etc.^30,41,42^. Bi_2_Te_3_ thin films grown on off-cut substrates would be an ideal system to explore such effect. The epitaxial growth transfers the texture of the substrate to the surface of the Bi_2_Te_3_, leading to systematically arranged atomic steps that can scatter the TSSs^21^.

In summary, we have theoretically proposed a new way to generate spin polarization and non-equilibrium spin current in a 3D TI by introducing the symmetry-breaking elastic scattering. The resulting new HPC component originates from the out-of-plane spin texture of the TSSs. This new HPC component provides a novel means for generation and manipulation of the out-of-plane spin polarized photocurrent in 3D TIs, offering new perspectives for spintronic and opto-spintronic applications exploiting the extraordinary properties of spin-momentum locking of the 3D TIs.

## Supplementary information

Supplementary information
