# Supplementary material for: Scattering symmetry-breaking induced spin photocurrent from out-of-plane spin texture in a 3D topological insulator
Source: Sci Rep. 2020 Jun 30;10:10610. doi: 10.1038/s41598-020-67612-3 (PMC7327057; doi:10.1038/s41598-020-67612-3)
Supplement: Supplementary file 1 — Supplementary information [file 41598_2020_67612_MOESM1_ESM.pdf]

Supplementary Information:

Scattering symmetry-breaking induced spin photocurrent from out-of-plane spin texture in a 3D topological insulator

Y. Q. Huang*, I.A. Buyanova, W.M. Chen

Department of Physics, Chemistry and Biology, Linköping University, S-581 83 Linköping, Sweden

**Correspondence to yuqing.huang@liu.se*

Supplementary Note 1: Selection of TSSs for the dynamic equation

To simulate CPGE in Bi_2Te_3, we only include the TSSs localized on the top surface in the dynamic equation. Such configuration breaks the inversion symmetry as required by CPGE and can qualitatively simulate the experimental condition under which the excitation light is absorbed and attenuated before reaching the bottom surface. The calculations are done on a k-point mesh generated in the 2D k-space area shown in Fig.2(b-c) according to the translation vector $b_1 = (-\frac{2\pi}{3a}, -\frac{2\pi}{\sqrt{3}a})/n_k$ and $b_2 = (\frac{2\pi}{3a}, -\frac{2\pi}{\sqrt{3}a})/n_k$. The convergence of the calculations is safely reached for k-mesh density with $n_k = 200$. We note that our choice of the k-points preserves the full symmetry of the crystal and thus avoids the risk of artifacts due to uncompensated current contributions from asymmetric distributed k-points. For a given $\boldsymbol{k}$, the target TSS is identified by requiring over 70% of the wavefunction to be located at the 1 QL on the top or bottom surface of the film [1]. A simple test is performed by allowing the optical transitions between the bulk states and TSS on both surfaces. This restores the inversion symmetry and the CPGE is found to vanish for all combinations of θ and ϕ. The motivation behind the choice of TSSs is to reduce the number of states involved in $\rho_{\boldsymbol{k}}^{\sigma\pm}$ and hence the computation time. Moreover, we note that CPGE is expected to be dominated by TSS for the following reasons. First of all, the CPGE originates from the spin-sensitive optical transitions which heavily rely on spin splitting of the band structure [2]. In materials with preserved bulk inversion symmetry, like Bi_2Te_3, CPGE is limited to the surface region where structure inversion symmetry breaks. This suggests a larger contribution of surface states as compared with the bulk states. Secondly, as shown by the earlier time-resolved ARPES results, the carrier lifetime of the TSS is considerably longer than the bulk state in Bi_2Te_3 [3]. While the photogenerated carriers in the bulk states are fully relaxed within 1ps, the non-equilibrium TSS population persists over a time range longer than 10 ps. The long

relaxation time has been attributed to the weak electron-phonon coupling associated with the TSS [3-5]. The long-lived photogenerated carriers in the Dirac cone would further suggest a dominant photocurrent contribution from the TSS. Finally, since the time-reversal symmetry protects the TSS from backscattering, the elastic scattering rate of TSS is strongly suppressed and, consequently, the carrier imbalance generated optically is better preserved than the bulk states.

Supplementary Note 2: scattering from other types of defects

In the main text, we concentrate on scattering from either short-range point or line defects for simplicity. However, the concerned symmetry consideration should generally apply to other types of defects. The scattering from a screened Coulomb potential is considered using the two-dimensional Thomas-Fermi model with the Fourier transform of the potential of the form, $\tilde{V}(\boldsymbol{k}) = \frac{2\pi e^2}{\varepsilon_r \varepsilon_0} \frac{e^{-kd}}{k + k_{TF}}$ [6]. Here, k_{TF} is the screening wavevector which is assumed to be 1 nm^{-1} for simplicity. $\varepsilon_r = 20$ is the dielectric constant [7]. d is the distance from the defect center to the surface of the Bi_2Te_3 and is assumed to be 1 nm. The scattering matrix is computed as usual following Eq. (5). The results of the k-space distribution of $n_{\boldsymbol{k}}$ and the incidence angular dependence of HPC calculated assuming a concentration of $10^9 cm^{-2}$ for the screened Coulomb defects are shown in Supplementary Fig. S3 (a) and (b), respectively. We also calculated the effect induced by scattering from a spin-orbit defect which is considered to be important for the transport behavior of 3D TI [8]. The scattering potential takes the form $\tilde{V}\left(\boldsymbol{k}'', \boldsymbol{k}'\right) = V_0 \lambda (\boldsymbol{k}'' \times \boldsymbol{k}') \cdot \boldsymbol{\sigma}$ with the simplification of a constant V_0. $\lambda = 2/k_F^2$, where k_F is the Fermi wavevector. $\boldsymbol{k}''$ and $\boldsymbol{k}'$ are the scattered and initial wavevector. Taking the TSS eigen state basis, the scattering rate is conveniently evaluated by $W_{\boldsymbol{k},\boldsymbol{k}'} = \frac{2\pi}{\hbar} n_i V_0^2 \lambda^2 [(\boldsymbol{k}'' \times \boldsymbol{k}') \cdot \hat{z}]^2 |\langle \boldsymbol{k}'' | \sigma_z | \boldsymbol{k}' \rangle|^2 \delta(E_{\boldsymbol{k}''} - E_{\boldsymbol{k}'})$ and the calculated results for $n_{\boldsymbol{k}}$ and HPC are shown in Supplementary Fig. S3 (c) and (d) for the same n_i and V_0 used for the short range defects. In both cases, the C_{3v} symmetry of the Bi_2Te_3 surface is preserved and, as a consequence, the scattering induced HPC vanishes as evident from the symmetric distribution of $n_{\boldsymbol{k}}$ and zero HPC under the normal incidence condition. Nevertheless, the $n_{\boldsymbol{k}}$ with the screened Coulomb scattering does exhibit a noticeable difference from other defects, which suggests a kinetics difference induced

by detailed scattering potential. However, the scattering induced HPC only appears after the symmetry is reduced.

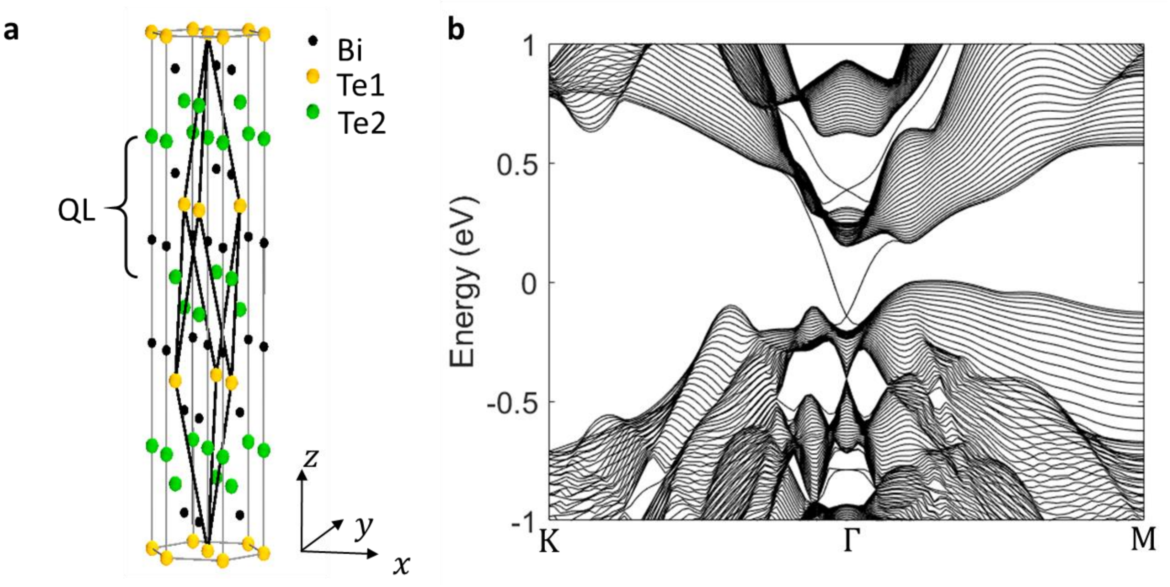

Supplementary Figure S1 (**a**) Unit cell of Bi_2Te_3 with the z axis aligned in the [111] direction. The QL is indicated. (**b**) The calculated energy dispersion of a Bi_2Te_3 thin film with 30 QLs. The dispersion relation is plotted along the K−Γ−M direction.

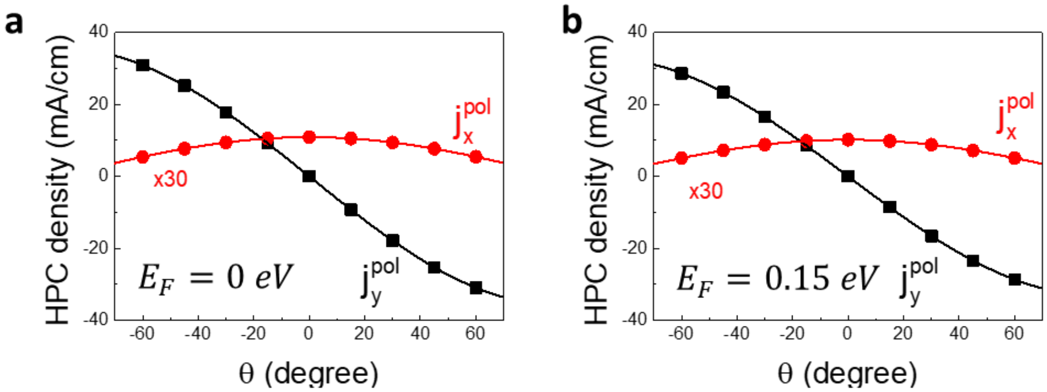

Supplementary Figure S2 (**a**) and (**b**) are the incidence angular dependence of HPC when E_F intersects the valence band ($E_F = 0\ eV$) and conduction band ($E_F = 0.15\ eV$), respectively, in the presence of elastic scattering from a line defect with a concentration

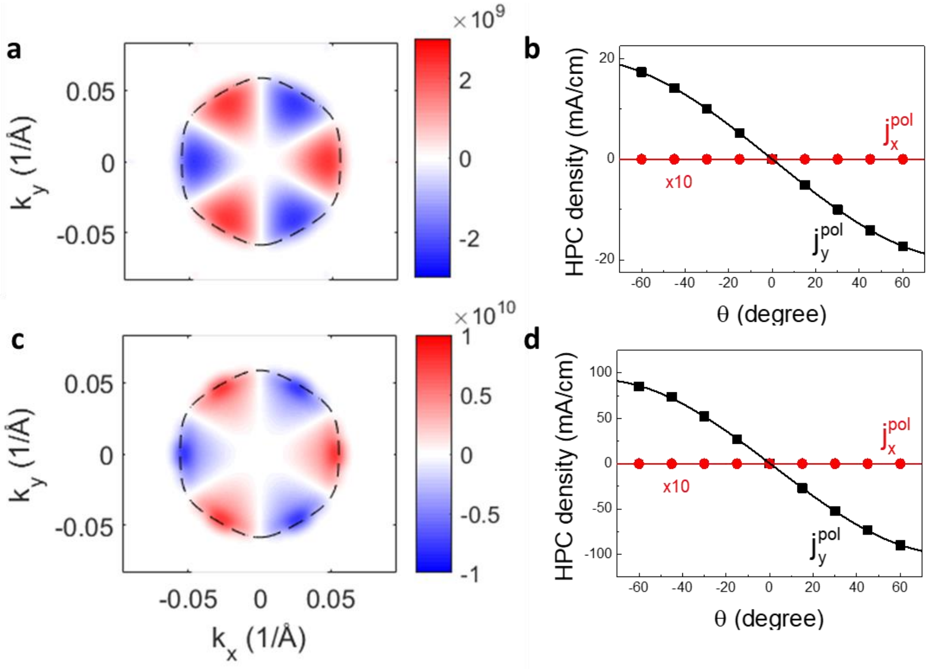

Supplementary Figure S3 (**a**) and (**b**) are, respectively, the $n(\boldsymbol{k})$ and incidence angular dependence of HPC calculated by involving the elastic scattering from the screened Coulomb potential. (**c**) and (**d**) are the $n(\boldsymbol{k})$ and incidence angular dependence of HPC calculated by including the spin-orbit scattering. The calculation parameters are described in details in the Supplementary Note 2.

References

1. Pertsova, A. & Canali, C. M. Probing the wavefunction of the surface states in Bi_2Se_3 topological insulator: a realistic tight-binding approach. *New J. Phys.* **16**, 063022; 10.1088/1367-2630/16/6/063022 (2014).
2. Ganichev, S. D. & Prettl, W. Spin photocurrents in quantum wells. *Journal of Physics: Condensed Matter* **15**, R935–R983; 10.1088/0953-8984/15/20/204 (2003).

3. Hajlaoui, M. *et al.* Ultrafast surface carrier dynamics in the topological insulator Bi_2Te_3. *Nano Lett.* **12**, 3532–3536; 10.1021/nl301035x (2012).

4. Sobota, J. a. *et al.* Ultrafast optical excitation of a persistent surface-state population in the topological insulator Bi_2Se_3. *Phys. Rev. Lett.* **108**, 117403; 10.1103/PhysRevLett.108.117403 (2012).

5. Pan, Z.-H. *et al.* Measurement of an exceptionally weak electron-phonon coupling on the surface of the topological insulator Bi_2Se_3 using angle-resolved photoemission spectroscopy *Phys. Rev. Lett.* **108**, 187001; 10.1103/PhysRevLett.108.187001 (2012).

6. Adam, S. & Das Sarma, S. Boltzmann transport and residual conductivity in bilayer graphene. *Phys. Rev. B* **77**, 115436; 10.1103/PhysRevB.77.115436 (2008).

7. Adam, S., Hwang, E. H. & Das Sarma, S. Two-dimensional transport and screening in topological insulator surface states. *Phys. Rev. B* **85**, 235413; 10.1103/PhysRevB.85.235413 (2012).

8. Adroguer, P., Liu, W. E., Culcer, D. & Hankiewicz, E. M. Conductivity corrections for topological insulators with spin-orbit impurities: Hikami-Larkin-Nagaoka formula revisited. *Phys. Rev. B* **92**, 241402; 10.1103/PhysRevB.92.241402 (2015).
